# FcRn mediated IgG transport by retinal pigment epithelium cells

**DOI:** 10.1186/1479-5876-10-S3-P2

**Published:** 2012-11-28

**Authors:** Nicoline M Korthagen, Kiki van Bilsen, Willem A Dik, Jeroen Bastiaans, Marion Kolijn, Robert W Kuijpers, Martin van Hagen

**Affiliations:** 1 Dept. of Immunology, Erasmus University Medical Center, Rotterdam, the Netherlands; 2 Dept. of Internal Medicine, Erasmus University Medical Center, Rotterdam, the Netherlands; 3 Rotterdam Eye Hospital, Rotterdam, the Netherlands; 4 Dept. of Ophthalmology, Erasmus University Medical Center, Rotterdam, the Netherlands

## Background

The neonatal Fc receptor (FcRn) binds immunoglobulin G (IgG), protects it from degradation and can transport IgG from apical to basolateral side of epithelial cells [1]. Previously, we showed that human retinal pigment epithelial (RPE) cells express FcRn and that, in contrast to non-ocular cells, TNF-α downregulates FcRn expression in RPE cells [2]. We speculated that FcRn in RPE plays a pivotal role in ocular IgG metabolism. The aim of this study was to develop a method to investigate whether RPE cells transport IgG molecules.

## Materials and methods

Monolayers of RPE cells, ARPE-19 (immortalized cell line) and human primary RPE cells (both fetal and adult), were established on Transwell culture inserts using fibronectin coated membranes. Trans-epithelial resistance (TER) measurements were used to evaluate monolayer formation. Plates with an average TER >35Ω*cm^2^ were used for subsequent experiments. Monoclonal IgG was added either apically or basolaterally and after incubation IgG was measured in the culture supernatant using an ELISA that was developed inhouse. To distinguish leakage of IgG through the cell layer from active transport by the cells we used NH_4_Cl or Chloroquine to block cytoplasmic vesicles.

## Results

The highest TERs were observed with ARPE-19 cells, while TERs often remained low with the primary cell cultures. The IgG level that crossed the membrane correlated positively incubation time, but this association was not linear. The observed effects of NH_4_Cl and Chloroquine were limited.

## Conclusions

Further experiments are needed to determine whether there is active transport by the RPEs. Paracellular diffusion will be investigated using dextran and specific blocking antibodies for the FcRn. In addition, experiments will be performed with RPE cells where FcRn is overexpressed or knocked-out.
